# An Elderly Case of Minimal Change Nephrotic Syndrome: Correlation between Renal Tubular Dysfunction and the Onset of Oliguric Acute Kidney Injury Requiring Hemodialysis

**DOI:** 10.1155/2024/1505583

**Published:** 2024-04-30

**Authors:** Maika Gojo, Chikayuki Morimoto, Syuntaro Taira, Minoru Yasukawa, Shinichiro Asakawa, Michito Nagura, Shigeyuki Arai, Osamu Yamazaki, Yoshifuru Tamura, Shigeru Shibata, Yoshihide Fujigaki

**Affiliations:** Department of Internal Medicine, Teikyo University School of Medicine, Itabashi-ku, Tokyo, Japan

## Abstract

Several theories have been proposed to explain the development of severe acute kidney injury (AKI) in patients with minimal change nephrotic syndrome (MCNS), but the exact mechanism remains unclear. We encountered an elderly patient with biopsy-proven MCNS who suffered from oliguric AKI, which required hemodialysis at the onset and during the first relapse of nephrotic syndrome. Throughout her relapse, we were able to monitor tubular injury markers, namely, urinary N-acetyl-*β*-D-glucosaminidase and urinary alpha-1-microglobulin levels. This patient had hypertension. 8.5 years after achieving complete remission, she experienced a relapse of nephrotic syndrome accompanied by AKI, necessitating hemodialysis. The hemodialysis was discontinued after 7 weeks of corticosteroid therapy and cyclosporin A treatment. During this relapse, we observed a correlation between the sudden increase in renal tubular injury markers and proteinuria levels and the progression of severe AKI. Conversely, a reduction in renal tubular injury markers and proteinuria was associated with the resolution of AKI. The abrupt elevation of both tubular injury markers and proteinuria levels suggests a possible breakdown in protein endocytosis in proximal tubular cells. Moreover, it is less likely that the acute reduction in intra-glomerular pressure is the primary cause of tubular injury, as it might result in a decrease in both glomerular filtration rate and proteinuria levels. It is conceivable that massive proteinuria, in conjunction with the patient's clinical characteristics, may contribute to tubular injury, ultimately leading to severe AKI in this patient.

## 1. Introduction

Acute kidney injury (AKI) is not uncommon in adult patients with minimal change nephrotic syndrome (MCNS) [1]. MCNS patients with AKI are characterized by increased urinary protein excretion, reduced serum albumin, older age, increased body weight, and hypertension at presentation compared with patients without AKI [2, 3]. A small number of MCNS patients have been reported to develop severe AKI, with or without the need for dialysis, and some of them may experience irreversible renal dysfunction [2]. Several factors have been suggested as contributors to the development of severe AKI in MCNS, encompassing ischemic renal injury [4, 5], tubular obstruction resulting from surrounding interstitial edema [6, 7], redistribution of renal blood flow from cortical to juxtaglomerular nephrons [8], decrease in capillary filtration coefficient (Kf) [9, 10], and tubular cell injury and apoptosis induced by massive proteinuria [11–13]. In fact, the pathophysiology of AKI that occurs in this setting is not clear and cases with oliguria requiring weeks and even months of dialysis still are poorly understood [1].

In this report, we present the case of an elderly woman with MCNS and AKI, requiring hemodialysis at both the onset and the first relapse. Throughout the clinical course of her nephrotic syndrome relapse with severe AKI, we had the opportunity to monitor biomarkers of tubular injury, such as urinary N-acetyl-*β*-D-glucosaminidase (uNAG) and urinary *α*1-microglobulin (u*α*1MG) levels. We observed a correlation between a sudden increase in biomarkers of tubular injury and the onset of severe AKI. Our discussion delves into potential mechanisms of severe AKI, focusing on tubular cell injury due to massive proteinuria in our patient.

## 2. Case Presentation

**Figure 1:**
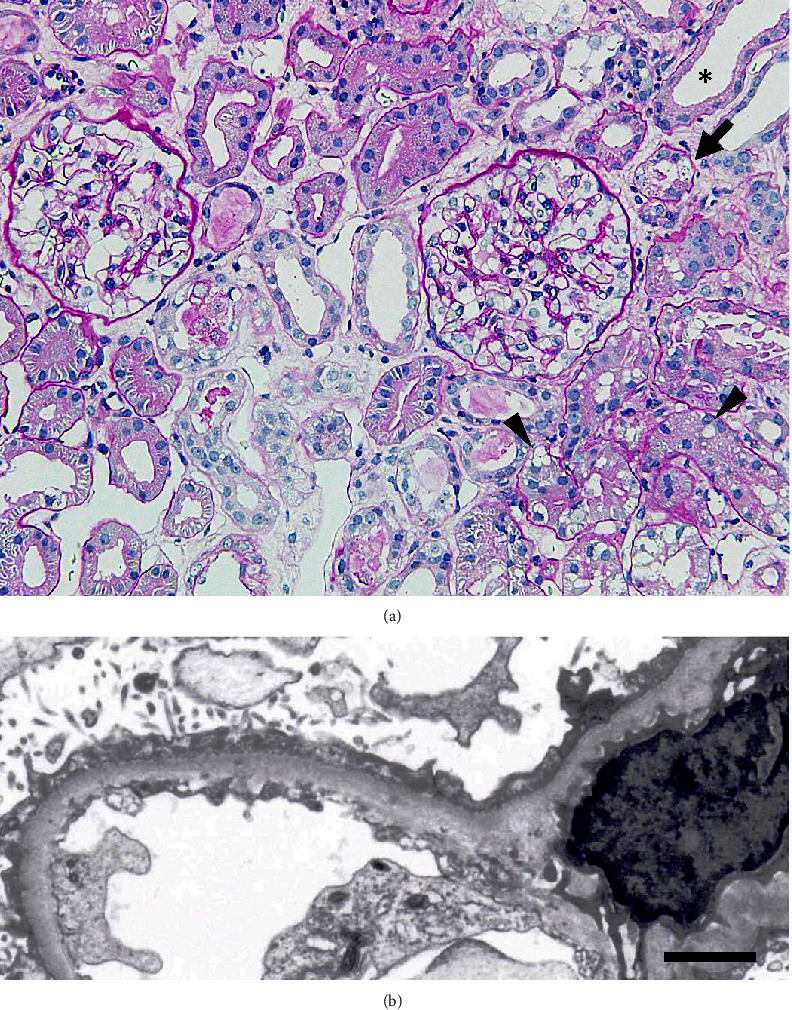
The kidney biopsy findings. (a) Light microscopy shows minor glomerular abnormalities, mild interstitial edema, and vacuolar regeneration in some proximal tubular cells (arrow heads), a necrotic renal tubule (arrow), and tubular simplification (asterisk). Original magnification ×200. Periodic acid-Schiff. (b) Electron microscopy shows diffuse foot process effacement and microvillous transformation of podocytes. Bar = 2.0 *μ*m.

A 75-year-old woman was admitted to our hospital due to a relapse of nephrotic syndrome. She had a medical history of hypertension at the age of 48, left breast cancer at the age of 49, and right breast cancer at the age of 52. She received a diagnosis of MCNS and severe AKI at the age of 66. At that time, she was taking 25 mg/day of atenolol. The laboratory data at the onset of the nephrotic syndrome are listed in Table 1. The kidney biopsy revealed minor glomerular abnormalities, mild interstitial edema, and vacuolar regeneration in some proximal tubular cells and a few necrotic renal tubules (Figure 1(a)). Tubular simplification, including loss of brush border of the proximal tubule and dilated tubule with flattening of tubular epithelium, was also observed (Figure 1(a)). No immune reactant depositions were found in the immunofluorescence study, while electron microscopy showed diffuse foot process effacement (Figure 1(b)).

**Figure 2:**
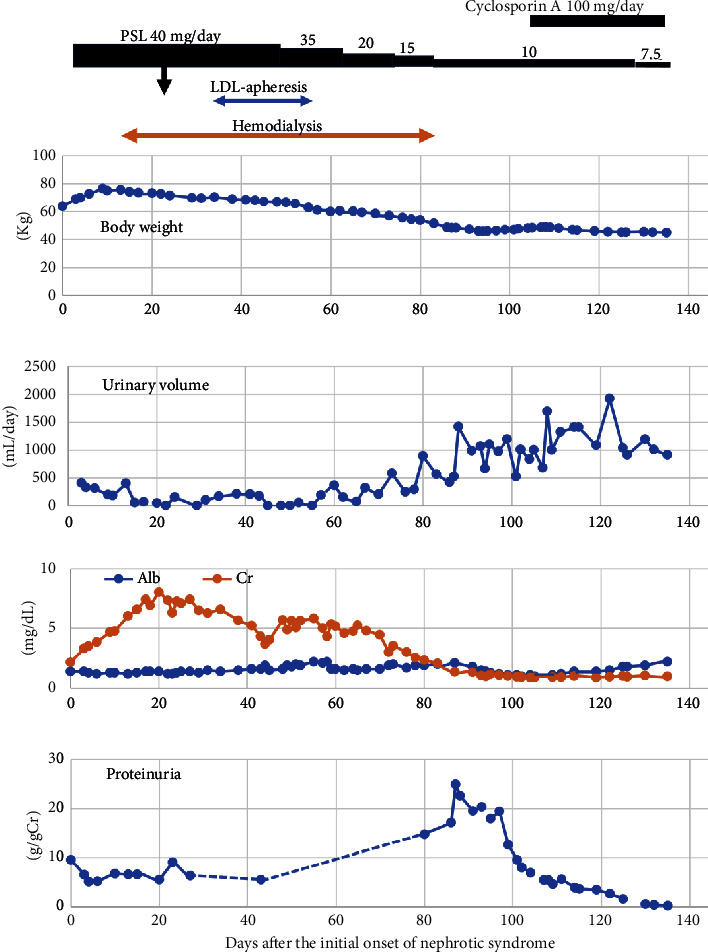
The clinical course after the onset of nephrotic syndrome. Arrow: steroid pulse therapy (500 mg of methylprednisolone given daily for 3 days); PSL: prednisolone; LDL-apheresis: low-density lipoprotein-apheresis; Alb: albumin; Cr: creatinine.

Her clinical course is depicted in Figure 2. She was initially treated with 40 mg/day of oral prednisolone, but she soon required hemodialysis due to oliguric AKI. Steroid pulse therapy (500 mg of methylprednisolone given daily for 3 days) was added. In consideration of possible focal segmental glomerulosclerosis with high low-density lipoprotein (LDL) cholesterol concentration, LDL-apheresis was performed. Unfortunately, she also developed intravascular catheter-related bacteremia and severe hypogammaglobulinemia (300 mg/dL). Alongside antibiotic therapy, prednisolone was started to be tapered. After 8 sessions of LDL-apheresis, it was halted due to thrombocytopenia and subcutaneous hemorrhage. Although recent reports suggest the efficacy of LDL-apheresis in patients with AKI and MCNS on hemodialysis, contributing to improved hypercoagulability and renal hemodynamics [14], our case did not align with these findings.

With a dose of 15 mg/day of prednisolone, her urinary volume began to increase in association with a reduction in serum creatinine (sCr). Eleven weeks after the initiation of hemodialysis, it was discontinued while she was on 10 mg/day of prednisolone. As nephrotic range proteinuria persisted, cyclosporin A was added at a dose of 100 mg/day. Subsequently, complete remission of nephrotic syndrome was achieved, and prednisolone and cyclosporin were tapered off 3 years and 9 months and 3 years and 3 months after starting treatment, respectively. sCr remained around 1.0 mg/dL. Her blood pressure was within the normal range without medication, but at the age of 74, she was prescribed 20 mg/day of telmisartan for high blood pressure.

At the age of 75, she presented with a one-week history of leg edema, a 4 kg increase in body weight, and reduced urine volume. She was diagnosed with a relapse of nephrotic syndrome and AKI and was subsequently admitted to our hospital. Upon admission, her blood pressure measured 137/96 mm·Hg, and her weight was 64 kg. Her sCr level was 0.95 mg/dL (estimated glomerular filtration rate (GFR) of 43.9 ml/min/1.73 m^2^), and proteinuria was 0.08 g/gCr, indicating chronic kidney disease stage 3bA1, noted 2 months prior to the relapse. Laboratory results are detailed in Table 1, with urine protein at 4.3 g/gCr, serum albumin at 1.6 g/dL, and sCr at 1.58 mg/dL. No significant elevation of inflammatory or autoimmune markers was observed (Table 1). Her cardiothoracic ratio (CTR) on chest X-ray was 54.5%, and inferior vena cava diameter on cardiac ultrasound was 14 mm with respiratory fluctuations.

A computed tomography scan revealed no cardiomegaly, mild pleural effusion, mild ascites, and normal-sized kidneys. The decision to conduct a kidney biopsy was approached with caution because of elevated D-dimer levels, indicating a possible requirement for anticoagulation therapy in the near future. Consequently, heparin was initiated.

**Figure 3:**
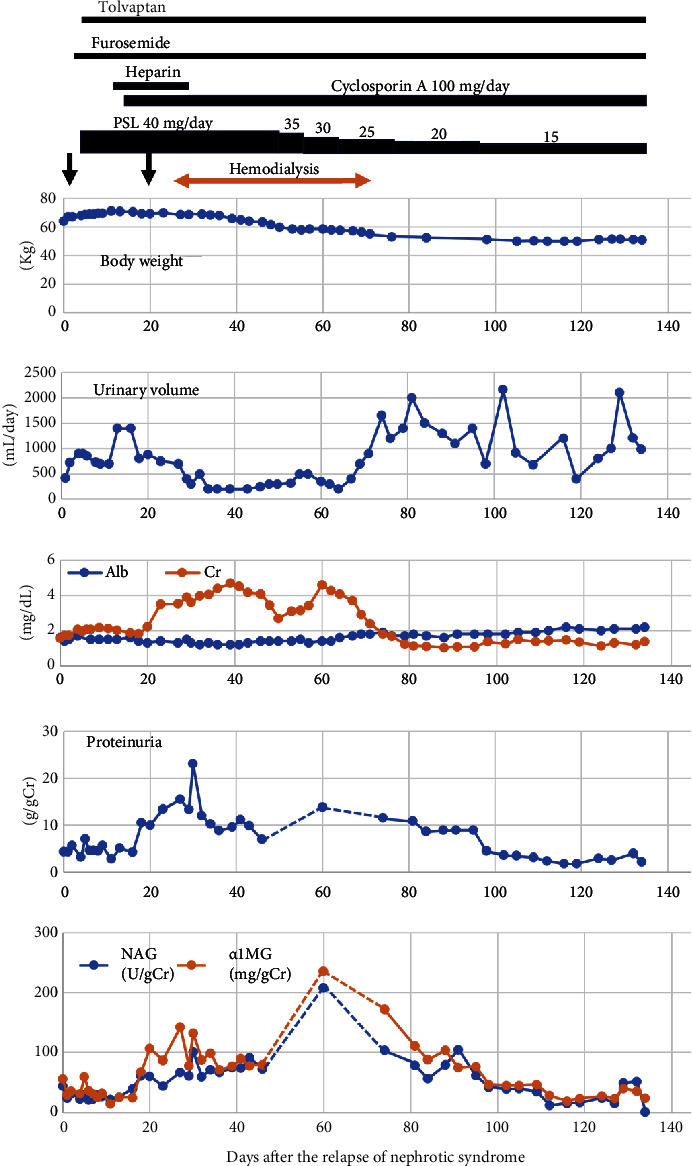
The clinical course after the relapse of nephrotic syndrome. Arrow: steroid pulse therapy (500 mg of methylprednisolone given daily for 3 days); PSL: prednisolone; Alb: albumin; Cr: creatinine; NAG: urinary N-acetyl-*β*-D-glucosaminidase; u*α*1MG: urinary *α*1-microglobulin.

Her clinical course is illustrated in Figure 3. After stopping telmisartan, she received intravenous furosemide and underwent steroid pulse therapy (500 mg of methylprednisolone, administered daily for 3 days), along with starting oral prednisolone at a daily dose of 40 mg. Tolvaptan was also introduced to enhance diuresis. On the thirteenth day of treatment, she began taking 100 mg/day of cyclosporin A. Despite her body weight peaking with a CTR increase to 58.2% on the 13th day of admission, her proteinuria, as well as uNAG and u*α*1MG, suddenly increased on the 18th day of admission. Subsequently, her sCr levels rose, leading to the initiation of hemodialysis through a vascular catheter.

Her hypogammaglobulinemia became severe, with levels falling below 200 mg/dL. As a result, prednisolone tapering was initiated 51 days after the treatment began. On the seventieth day of admission, her urine volume began to increase, and hemodialysis was discontinued seven weeks after its initiation. She continued to exhibit nephrotic-range proteinuria, but both proteinuria and renal dysfunction gradually improved without any additional treatment. At her last follow-up, her urinary protein excretion was 2.15 g/gCr, and sCr was 1.36 mg/dL. She was discharged to a rehabilitation hospital.

## 3. Discussion

In our current case, the evidence of prerenal AKI based on fractional excretion of sodium was apparent. However, assessments of CTR, inferior vena cava diameter, hematocrit, N-treminal pro-B-type natriuretic peptide, and plasma renin activity (Table 1) did not reveal significant intravascular volume depletion. This finding aligns with the overfill hypothesis of sodium and water retention in nephrotic syndrome [15]. Despite differences in the AKI phase between the onset and relapse of nephrotic syndrome in our case (Table 1), unlike scenarios where renal tubular obstruction leads to AKI due to interstitial edema, the peak of body weight gain did not coincide with a sudden reduction in renal function. Our prior research unveiled elevated levels of uNAG and u*α*1MG in both AKI and non-AKI patients with MCNS at the time of kidney biopsy, with significantly higher levels in the AKI group [16]. These elevated levels were positively correlated with proximal tubular injury, as indicated by vimentin expression [16]. This led us to hypothesize that severe proteinuria triggers specific tubular insults, predisposing adult MCNS patients to severe AKI.

Although it is well known that tubular injury markers are elevated in MCNS patients with AKI requiring hemodialysis, the relationship between the development of tubular injury and the decline in GFR remains unclear. In our MCNS patient, we had the opportunity to monitor the time-sequential changes in uNAG and u*α*1MG levels throughout the clinical course of severe AKI development and resolution. It has been reported that u*α*1MG exceeding 15 mg/gCr and uNAG levels higher than 15.0 U/gCr strongly suggest proximal tubular dysfunction due to failure to reabsorb low-molecular-weight proteins and excessive NAG enzyme discharge, respectively [17, 18]. While the reliability of tubular injury markers and proteinuria by urinary creatinine ratio in AKI is questionable, the continuous changes in uNAG and u*α*1MG values in our patient suggest the potential for evaluating their rise and fall. In our patient, both u*α*1MG and uNAG levels were moderately elevated at the onset of nephrotic syndrome and during a relapse before hemodialysis initiation. Throughout the relapse, a suddenly severe increase in uNAG and u*α*1MG levels coincided with a rapid rise in urinary total protein excretion. This increase was followed by a sudden elevation in sCr levels, ultimately necessitating hemodialysis.

Generally, a reduction in GFR leads to a decrease in urinary protein excretion. A study by Arisz et al. demonstrated that in patients with nephrotic syndrome, a decrease in GFR induced by indomethacin administration resulted in a greater decrease in urinary protein excretion than the reduction in GFR [8]. Renin-angiotensin system (RAS) inhibitors, known to reduce intra-glomerular pressure, may also lead to a notable decrease in urinary protein levels when MCNS is complicated by RAS inhibitor-associated AKI [19]. Reduced intra-glomerular pressure may explain severe AKI in MCNS, but it does not account for the abrupt increase in sCr levels observed in our patient, correlating with increased urinary total protein excretion. Thus, intra-glomerular hemodynamic changes may not be the primary cause of the abrupt reduction in GFR in our patient.

It is now recognized that increased urinary protein concentrations result from increased permeability of the glomerular basement membrane and impaired reabsorption in proximal tubules [20, 21]. In our case, the abrupt rise in both tubular injury markers and total proteinuria levels suggests, in addition to increased permeability of the glomerular basement membrane, a possible disruption in protein endocytosis in proximal tubular cells [17, 22]. This phenomenon may be interpreted as massive proteinuria leading to proximal tubular injuries and renal dysfunction [11–13]. Notably, it has been reported that both autophagosomes and lysosomes increase in patients with MCNS who have proteinuria [23]. Experiments with the human proximal tubule epithelial cell line, HK-2, treated with urinary proteins, also showed lysosomal membrane permeabilization and dysfunction [13, 23]. Furthermore, lysosomal enzymes released into the cytoplasm can trigger proximal tubule epithelial cells to release inflammatory factors, leading to apoptosis, and in severe cases, even necrosis [13, 24].

It has been suggested that elevated excretion of uNAG and urinary low molecular weight proteins, such as urinary retinol-binding protein, urinary *β*2-microglobulin (u*β*2MG), or urinary lysosome, may serve as markers of proximal tubular dysfunction in various nephrotic syndromes, including MCNS, focal segmental glomerulosclerosis, or mesangial proliferative glomerulonephritis, which often characterize corticosteroid-resistant patients [25–27]. u*β*2MG is commonly utilized in clinical settings to assess proximal tubular function. However, it is known and we experienced that u*β*2MG is unstable in acidic urine with a pH of 6 or less, and values from samples with a pH of 6 or less may be highly misleading for u*β*2MG [28]. Therefore, we refrained from using u*β*2MG levels to evaluate tubular injury in our case. Further investigation is necessary to confirm whether the extent of uNAG and urinary low molecular weight protein excretion has predictive value for renal functional outcomes and response to corticosteroid therapy in MCNS patients.

Tubular dysfunction in AKI may also manifest as electrolyte disturbances, such as urinary electrolyte wasting (potassium, magnesium, and phosphate) [29, 30]. In our patient, dilutional hyponatremia was evident during the second presentation; however, no findings suggestive of urinary electrolyte wasting were observed (data not shown).

In summary, our patient's clinical course supports the idea that tubular injury due to massive proteinuria in conjunction with the patient's clinical characteristics is a crucial mechanism underlying the development of severe AKI in MCNS. However, uNAG and u*α*1MG increased abruptly and concurrently with the decline in GFR in our patient, making their spot measurements challenging for predicting severe AKI development. Other more sensitive markers of tubular injury, such as neutrophil gelatinase-associated lipocalin, kidney injury molecule-1, and liver-type fatty acid binding protein, may prove to be more effective for prediction purposes.

## Figures and Tables

**Table 1:** Laboratory data at the onset and the relapse of nephrotic syndrome and acute kidney injury.

	Onset	Relapse
*Urine*		
Protein (g/gCr)	4.7	4.3
NAG (U/L) (0.7 to 11.2)	100.1	88.9
NAG (U/gCr)	30.3	44.1
*α*1-microglobulin (mg/L) (1.0 to 5.0)	115.0	113.5
*α*1-microglobulin (mg/gCr)	34.9	56.4
*β*2-microglobulin (*μ*g/L) (0 to 150)	190	30>
*β*2-microglobulin (*μ*g/gCr)	57.6	25.6>

*Complete blood count*		
White blood cell (*μ*L)	6.500	10.700
Hematocrit (%)	41.7	41.7
Platelet (*μ*L)	21.6 × 10^4^	17.2 × 10^4^
D-dimer (*μ*g) (0 to 4.9)	3.9	5.9

*Blood chemistry*		
Albumin (g/dL)	1.4	1.6
Urea nitrogen (mg/dL)	57.5	38.4
Creatinine (mg/dL)	2.18	1.58
Estimated GFR (ml/min/1.73 m^2^)	18.4	25.2
LDL-C (mg/dL)	279	308
Na (mEq/L)	120	116
K (mEq/L)	4.6	4.8
Cl (mEq/L)	91	85
Ca (mg/dL)	7.2	7.5
P (mg/dL)	4.1	4.4
Mg (mg/dL)	2.3	2.0
NT-proBNP (pg/mL) (0–55)	897.8	1,573
Renin activity (ng/mL/hr) (0.2 to 2.3)		1.0
Aldosterone concentration (pg/mL) (4.0 to 82.1)		52.4

*Immunologic test*		
IgG (mg/dL)	385	476
IgA (mg/dL)	177	233
IgM (mg/dL)	164	193
IgE (IU/mL)	820	89
C-reactive protein (mg/dL)	0.01	0.07
Antinuclear antibody × (<40)	40>	40>
Anti-dsDNA antibody (IU/mL) (<9.0)	0.5	0.6>
MPO-ANCA (U/mL) (<3.4)	1.0	1.0
PR3-ANCA (U/mL) (<3.4)	1.0	1.0

*Index*		
FENa (%)	0.03	0.48

NAG: N-acetyl-*β*-D-glucosaminidase; GFR: glomerular filtration rate; LDL-C: low-density lipoprotein cholesterol; NT-proBNP: N-terminal pro-brain natriuretic peptide; MPO-ANCA: myeloperoxidase-anti-neutrophil cytoplasmic antibody; PR3-ANCA: proteinase 3-anti-neutrophil cytoplasmic antibody; GBM: glomerular basement membrane; FENa: fractional excretion of sodium. The values in the parentheses show the normal range.
